# Uniformity in Accounts

**Published:** 1891-01-24

**Authors:** 


### Passing Topics.

### UNIFORMITY IN ACCOUNTS.

We are glad to know that the Hospital Sunday Fund Council is at last moving in regard to a matter which has attracted at various times some attention, though never, perhaps, quite as much as it merits, or we should not have waited for action so long. The difficulty has been, and still is, we suppose, to get those concerned to agree upon common action of any kind, and to believe that everybody is anxious to see uniformity in anything in the hospital world would be to fall into grievous error. A disposition to resent interference, and a stubborn disinclination to surrender an iota of individual liberty, are characteristic of the government of nearly all hospitals. Independence is bred in the bone of voluntary institutions, and precisely as the individual subscriber claims the right to give as he pleases, when he pleases, and to whom he pleases, and is quick to resent a suggestion that his generosity might be more wisely exercised, so the managers are suspicious of every proposal which seems to savour of authority and control superior to their own. And in this matter of uniformity in the preparation of accounts we confess we can have no well-founded belief in its adoption until the hospitals show some sign of recognising that not isolation, but unity, is strength.

Of course, if an institution is able to be independent throughout, well and good. But this is what hospitals, supported by subscriptions, cannot afford to be. They must justify their work to a public opinion of some sort, and what is more important than that their accounts should be intelligibly prepared and made as far as possible capable of comparison—one institution with another? It is easy to answer, "we don't want comparisons, and we are not in the least concerned to satisfy those who do;" but hospital managers may rest assured of this, that the aggravated difficulties which threaten hospital finance in the future will not be surmounted by a refusal to recognise the causes from which they spring. We are disposed to resent as warmly as any the criticism of persons who are at no pains to assist the institution, and who, consequently, have acquired no constitutional right to interfere in its affairs. But, whether happily or unhappily, powers are frequently wielded by those who would have some difficulty to prove rightful possession, and in these days when nothing is held sacred it would be childish to expect our hospitals to be free from attack.

The curious discrepencies between the several items of expenditure presented by different institutions appear to invite observation and criticism, and if, as is the fact, managers themselves are at a loss to explain why the cost per bed in one hospital should differ so greatly from that in another, where the conditions appear similar, or why one hospital should expend per head upon a particular item double or treble the amount found sufficient elsewhere, how can we expect outside critics to be satisfied? Of course there will always be differences in detail, and considerable differences, too: the fact, say, of one general hospital getting a far larger proportion of surgical cases than another will at once constitute a radical difference in the incidence, and presumably, in the total of its expenditure; but this makes it all the more desirable that some fundamental methods of presenting the accounts should be agreed upon. So diverse are the notions, even among experts, that the calculation of the recorded cost per bed is, in some cases, arrived at by including, and sometimes by excluding, the expenditure upon out-patients. This one illustration ought to suffice to show that a common understanding is needed. We approve of the contemplated action of the Hospital Sunday Council, and are glad that in this instance, at any rate, the proposal for enquiry and discussion comes from a friendly, and not a hostile quarter. Agricola.

